# Prevalence of Chronic Diseases and Alterations of Gut Microbiome in People of Ningxia China During Urbanization: An Epidemiological Survey

**DOI:** 10.3389/fcimb.2021.707402

**Published:** 2021-11-03

**Authors:** Yong Du, Lu Ding, Li Na, Ting Sun, Xian Sun, Liqun Wang, Shulan He, Zhizhong Wang, Zhenhui Lu, Feng Li, Xiaofei Guo, Yanhong Zhang, Jin Tian, Bo Wang, Sifan Zhai, Chao Yang, Xiao Liu

**Affiliations:** ^1^ Biobank, Laboratory of Surgery, General Hospital of Ningxia Medical University, Yinchuan, China; ^2^ Surgical Laboratory, General Hospital of Ningxia Medical University, Yinchuan, China; ^3^ College of Clinical Medicine, Ningxia Medical University, Yinchuan, China; ^4^ Faculty of Health Sciences, University of Macau, Macau, China; ^5^ School of Public Health and Management, Ningxia Medical University, Yinchuan, China; ^6^ School of Public Health at Guangdong Medical University, Dongguan, China; ^7^ Center of Laboratory Medicine, General Hospital of Ningxia Medical University, Yinchuan, China; ^8^ Department of Ultrasound, General Hospital of Ningxia Medical University, Yinchuan, China; ^9^ Department of Nursing, General Hospital of Ningxia Medical University, Yinchuan, China

**Keywords:** urbanization, chronic diseases, gut microbiota, epidemiological investigation, microbiome

## Abstract

The continuous development of urbanization has dramatically changed people’s living environment and lifestyle, accompanied by the increased prevalence of chronic diseases. However, there is little research on the effect of urbanization on gut microbiome in residents. Here we investigated the relation between living environment and gut microbiota in a homogenous population along an urban-rural gradient in Ningxia China. According to the degree of urbanization, the population is divided into four groups: mountainous rural (MR) represents non-urbanized areas, mountainous urban (MU) and plain rural (PR) represent preliminary urbanization, and plain urban (PU) is a representative of complete urbanization. Studies have found that with the deepening of urbanization, the prevalence of chronic diseases, such as diabetes, dyslipidemia, fatty liver, gallstones, and renal cysts, have gradually increased. The intestinal richness and diversity of the microbial community were significantly reduced in the PR and the PU groups compared with the MR and the MU groups. Based on linear discriminant analysis selection, the significantly enriched genera Faecalibacterium, Prevotella, and Pseudobutyrivibrio in the MR group gradually decreased in the MU, the PR, and the PU groups. Effect size results revealed that both residence and diet had an effect on intestinal microbiota. Our results suggested that the disparate patterns of gut microbiota composition were revealed at different levels of urbanization, providing an opportunity to understand the pathogenesis of chronic diseases and the contribution of the “rural microbiome” in potential protection against the occurrence of chronic diseases.

## Background

Urbanization is used to describe the process of social transition from rural to urban lifestyles, including a gradual increase in the proportion of the population living in urban areas (Swinburn et al., 2011; Gong et al., 2012). Environmental exposure during urbanization, including westernization of diet, contamination, microbial exposure in early life, and improvement in hygiene have been shown to influence gut microbiome (Zuo et al., 2018; Sonnenburg and Sonnenburg, 2019). As a major demographic feature in the 21st century, urbanization has a great impact on the health of residents. Namely, the rapid urbanization experienced in developing countries is associated with increasing incidence of various forms of diseases, including diabetes, obesity and gastrointestinal diseases (Allender et al., 2008; Cheema et al., 2014; Kaplan and Ng, 2016).

The intestinal microecosystem is the largest microecosystem in the human body and plays an important role in human health and disease (Ley et al., 2006; Nenci et al., 2007). Accumulating evidence suggests that gut microbiota is a key factor in modulating the host immune system, influencing susceptibility to autoimmune diseases (Belkaid and Hand, 2014; Gensollen et al., 2016). Urbanization attenuates microbial diversity and might play a role in the pathogenesis of chronic diseases (Zuo et al., 2018; Sonnenburg and Sonnenburg, 2019). The effect of urbanization is likely to be reflected in human gut microbiome, as alterations in the gut microbiome have been associated with inflammatory and autoimmune diseases (Manichanh et al., 2012). However, to date, there is no direct evidence on how urbanization affects microbiome composition during urbanization. Some studies have assessed the impact of urbanization on the gut microbiome by comparing the gut microbiome of healthy individuals in rural and urban areas. It has been reported that people living in non-Western and/or rural areas have higher bacterial diversity compared to those in the United States and Europe (De Filippo et al., 2010; Yatsunenko et al., 2012; Schnorr et al., 2014; Martínez et al., 2015). Fecal bacteria from children in a rural African village in Burkina Faso have been reported to be similar to the microbiome of early human settlement at the time of the birth of agriculture (De Filippo et al., 2010). In addition, children from Burkina Faso, who consumed predominantly a high-fiber diet, had a unique abundance of bacteria of the genera *Prevotella* and *Xylanibacter*, which are known to contain a set of bacterial genes for cellulose and xylan hydrolysis (De Filippo et al., 2010). Similar results have been reported for a higher abundance of *Bacteroides*, *Bifidobacterium*, *Blautia* and *Dorea* in populations from highly urbanized regions of the United States and Europe, while rural populations from Africa and South America are enriched in *Prevotella* and members belonging to *Clostridiaceae* family (Yatsunenko et al., 2012; Ou et al., 2013; Schnorr et al., 2014; O’Keefe et al., 2015; Obregon-Tito et al., 2015; Gomez et al., 2016). Substantial differences were found in a study comparing fecal microbiota composition in African descendants living in rural, semi-urban areas to those living in urban areas, with *Prevotella* predominating in semi-urban individuals and *Bacteroides* predominating in African Americans (Zoetendal et al., 2013). A study of gut microbial communities from four Himalayan populations representing different survival strategies suggests that differences in gut microbiota from foraging populations are strongly associated with agricultural dependence in these populations (Jha et al., 2018). These studies compare the gut microbiota composition only in terms of geographical diversity. Our understanding of the role of gut bacteria in relation to factors associated with urbanization is still in its infancy. In particular, there are fewer studies comparing rural and urban microbiomes in homogeneous ethnic populations, and this type of study is more meaningful for us to examine the effects of urbanization on chronic diseases and intestinal microbiota.

**Figure 1:**
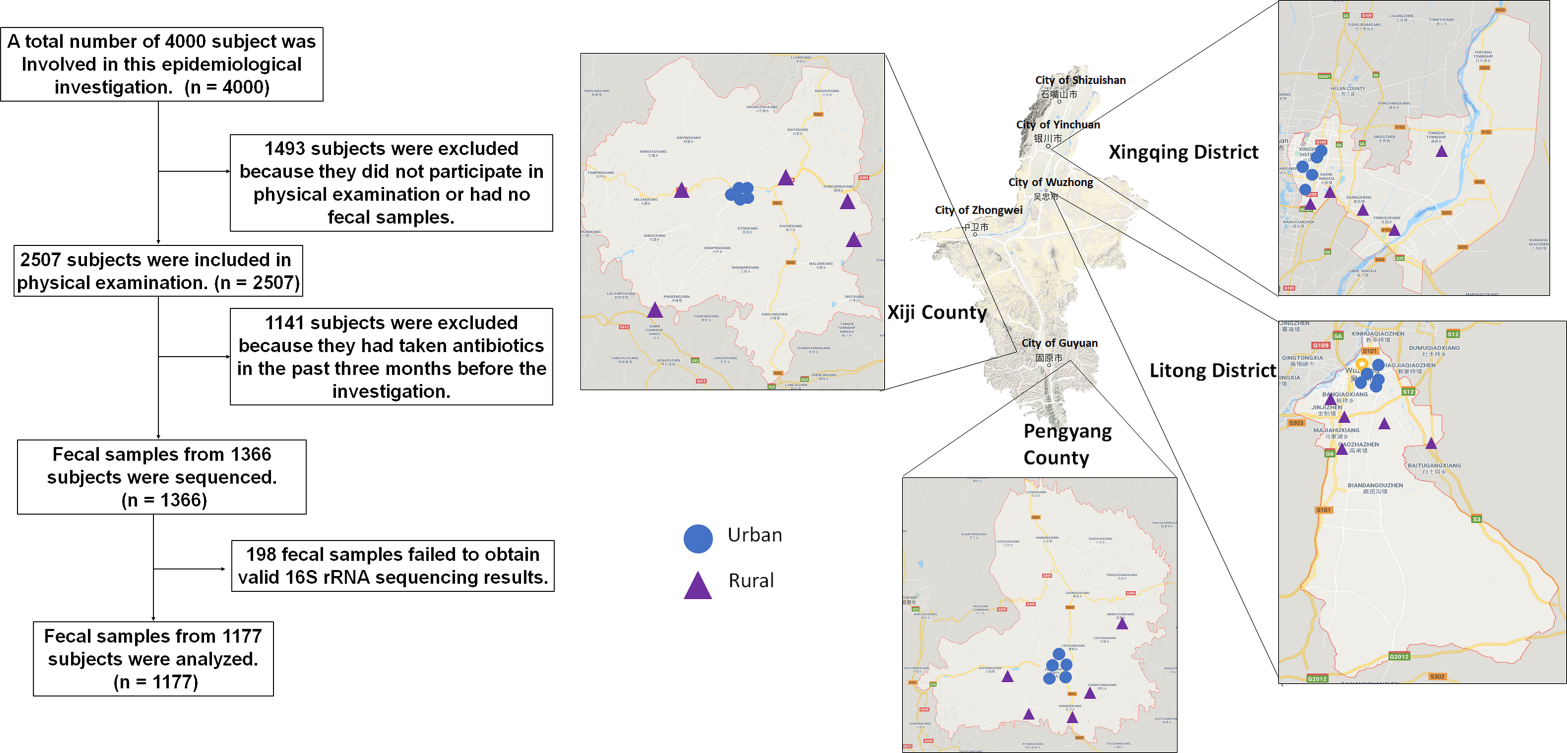
Screening Process and flow diagram.

Here, we performed a comprehensive study integrating multidimensional dataset of gut microbiota, and host characteristics that were based on clinical and questionnaire-derived data, from a large-scale of epidemiological investigation of 538 mountain rural (MR) participants, 529 mountain urban (MU) participants, 765 plain rural (PR) participants and 675 plain urban (PU) participants. The sample screening process is described in **Figure 1**. Since the gut microbial ecosystem reflects external environmental triggers, the study of the gut microbiota in residents during urbanization could shed light on the pathogenesis of chronic diseases and provide insights into the prevention of chronic diseases.

## Methods

### Geographical Regions and Subject Recruitment

This epidemiological survey was conducted in Ningxia Hui Autonomous Region, China, which lies between 35°14′ and 39°23′ north latitude and 104°17′ to 107°39′ east longitude. The shape of Ningxia is narrow and long, the topography in Ningxia is complex, and the elevation varies greatly. It lies on the mountainous plateau and crosses the plains and mountains (**Figure 1**).

Multi-stage sampling was used to select study subjects. By the end of 2018, there were 5 prefecture-level cities (9 municipal districts, 2 county-level cities, and 11 counties) in Ningxia Hui Autonomous Region. According to the economic development level and location distribution, Xingqing District, Litong District, Pengyang County, and Xiji County were selected as the survey sites in this study (**Figure 1**). Stratified in these four counties and districts according to urban-rural differences, five neighborhoods (or villages) were selected by simple random sampling at each level, 100 households were selected according to systematic sampling within each neighborhood (or village), and one study subject was selected by KISH table from each household for epidemiological investigation and physical examination (**Figure 1**). Participants must have lived at the survey site for more than 6 months and are aged 18-80 years old.

According to statistics in 2019, the resident population of Ningxia was 6,946,600. 4,000 people were sampled in this epidemiological survey, and 2,507 people finally participated in blood routine tests. According to the resident population, the participants in this survey can be divided into four groups: MR, MU, PR and PU. The topography and economic development status of the population illustrate the degree of urbanization: PU represents the most urbanized part of Ningxia, MR represents the unurbanized part, and MU and PR are in between.

### Disease Examination and Data Collection

The classification and diagnosis of diabetes were made according to the Medical Care Standard for Diabetes – 2019 (Association, 2018). The 2016 “Guidelines” recommend the appropriate levels and abnormal cutoff points for the blood lipid components in the Chinese population. The diagnostic criteria for dyslipidemia were: LDL-C > 3.4 mmol/L or TC > 5.2 mmol/L, HDL-C < 1.04 mmol/L or TG > 1.7 mmol/L (revision Jcfg, 2018). The 2018 Chinese hypertension guidelines did not follow suit with the US guidelines and maintained the current cut-point of ≥140/90 mmHg for hypertension diagnosis. Diffuse fatty liver can be defined by the presence of at least two of three abnormal findings on abdominal ultrasonography: diffusely increased liver near field ultrasound echo (‘bright liver’), liver echo greater than kidney; vascular blurring and the gradual attenuation of far field ultrasound echo. The typical findings of fatty liver in computed tomography (CT) include a diffuse decrease of liver density. Portal and hepatic vein branches appear prominent in a scan unenhanced with contrast. A -liver-to-spleen CT ratio less than 1 is defined as fatty liver: a mild degree of fatty liver has a liver: spleen CT ratio of less than 1 but more than 0.7; a moderate degree of fatty liver has a ratio of less than or equal to 0.7 but more than 0.5, and a severe degree of fatty liver has a ratio less than or equal to 0.5 (Farrell et al., 2007).

The epidemiological survey database was compiled by Ningxia Medical University using Epidata software. Before the investigation, students of preventive medicine related majors were trained to standardize operation methods. Data entry and on-site investigation were conducted simultaneously, and real-time verification of double entry was used. Each group had on-site management personnel for summarization and error correction. The questionnaire was checked daily, and the unqualified questionnaire was returned to the investigator for correction. Five percent of the questionnaires, blood samples, and ultrasonography results were sampled and reviewed. Qualified data are uploaded to the database and managed by the administrator.

The details of 2507 subjects recruited in the present study are provided in the **Tables 1** and **S1**. The entire study was reviewed and approved by the Institutional Review Board of the General Hospital of Ningxia Medical University (No. 2017-200). Informed consents on enrolment had been signed and provided by all participants. Demographic and clinical data of the participants were obtained from the hospital’s questionnaires and hospital electronic medical records.

**Table 1:** Clinical characteristics of participants in this study.

	MR N = 538	MU N = 529	PR N = 765	PU N = 675	p value
**Gender (Male)**	231 (42.94%)	179 (33.84%)	274 (35.82%)	232 (34.37%)	0.005
**Age**	53.0 [44.0-63.0]	49.0 [40.0-63.0]	53.00 [45.0-62.0]	55.0 [45.5-63.0]	<0.001
**Body mass index**	24.03 [22.03-26.43]	24.77 [22.51-26.90]	25.48 [23.35-27.78]	25.00 [22.43-27.44]	<0.001
**Diabetes**	32 (5.95%)	40 (7.56%)	69 (9.02%)	94 (13.93%)	<0.001
**Dyslipidemia**	156 (29.00%)	181 (34.22%)	517 (67.58%)	445 (65.93%)	<0.001
**Hypertension**	186 (35.09%)	209 (40.12%)	260 (34.48%)	189 (28.38%)	<0.001
**Fatty liver disease**	49 (9.11%)	112 (21.17%)	259 (33.86%)	231 (34.22%)	<0.001

MR, Mountain rural; MU, Mountain urban; PR, Plain rural; PU, Plain urban. Categorical data are presented as numbers with percentages in round parentheses and were tested using the chi-square test or Fisher’s exact test. Continuous data are presented as median and interquartile range in squared parentheses and were tested using the Kruskal-Wallis H test. P values < 0.05 were considered statistically significant.

In order to study the dietary habits in different regions and the potential effect of diet on intestinal bacteria, we conducted a questionnaire survey on the dietary status of participants. The survey included 20 pre-dispensed food types. For each category of food, participants were asked about their weekly consumption and converted to annual consumption.

### DNA Extraction and Sequencing

Selection criteria for participation in the gut microbiota test: (i) no antibiotics/antifungal/antiviral drugs in the past month, (ii) no chronic intestinal disease (IBD/inflammatory bowel syndrome/chronic diarrhea/abdominal tuberculosis). The fecal samples were kept at -80°C before extraction of genomic DNA. The DNA extraction was conducted as the method described in the literature (Godon et al., 1997). The quality and quantity of DNA were assessed using Bio spectrometer (Eppendorf, Germany) and 0.8% agarose gel electrophoresis. The 16S rRNA V3-V4 region was amplified by PCR using 341F (5’-CCTACGGGNGGCWGCAG-3’) and 805R (5’-GACTACHVGGGTATCTAATCC-3’) primers. PCR products were recovered using 2% agarose gels and purified using the AxyPrep DNA Gel Extraction Kit (Axygen Biosciences, Union City, CA, USA). The final 16S rRNA gene amplicon library was sequenced on the MiSeq platform (Illumina) using a 2 × 300 bp paired-end protocol. Illumina MiSeq sequencing was performed by Shanghai Mobio Biomedical Technology Co., Ltd. (China).

### Data Processing, OTU Clustering, and Taxonomic Profiling

Sequencing read pairs were demultiplexed based on the unique molecular barcodes, and reads were merged using USEARCH Version 8.0. Merging allowed 0 mismatches and a minimum overlap of 50 bases. Sequences that cannot be spliced and chimeras were removed, and chimeras were eliminated using UCHIME software. Sequences less than 400 bases in length after splicing were removed. Operational Taxonomy Units (OTUs) were clustered using UPARSE (Edgar, 2013) (version 7.1 http://drive5.com/uparse/) software based on 97% similarity. OTUs were determined by mapping the centroids to the SILVA v128 database. Other analyses were performed using the QIIME 1.9 pipeline (Caporaso et al., 2010).

Bacterial Diversity and Taxonomic Analysis using relative abundances of the OTUs and rarefied data: By a sampling-based OTUs analysis, bacterial diversity was determined and shown by the Ace index, the Chao 1 index, the Shannon index, and the Simpson index by using the “vegan” package in R (Oksanen et al., 2019). Principal coordinate analysis (PCoA) based on Bary-Curtis distance were conducted to display the microbiome space between both the group samples.

### Effect Size Analysis

In this study, we performed “effect size” analysis strategy to determine whether the clinical and dietary datasets can affect gut microbiota. To assess the proportion of variance of gut microbiota that be explained by clinical and dietary datasets, the adonis function of the R package vegan was used to estimate the “one-to-all” effect size (R^2^) between each variable of the clinical and dietary datasets to the whole gut microbiota dataset. Only variables with significant effect on the gut microbiota dataset were considered. To get rid of redundant variables, the spearman correlation coefficient between variables was calculated, and variables with correlation coefficient greater than 0.5 were removed.

### Functional Annotation of 16S rRNA Genes Based on KEGG Profile

In order to identify the differentially abundant functional processes and pathways across the four groups, predictive functional profiling was performed on the taxonomic profiles obtained according to the 16S rRNA gene amplicon sequencing using the PICRUSt version 1.0.0 pipeline (Langille et al., 2013).

### Statistical Analysis

PERMANOVA and ANOSIM were performed respectively using the adonis and the anosim functions implemented in the “vegan” package of R (Oksanen et al., 2019). Spearman Rank correlation test was performed to analyze the correlation between the clinical variables and the bacterial genera in stool. Principal component analysis (PCA) analysis of clinical and dietary variables was performed using the prcomp function in R. We performed PERMANOVA to study the differences in the bacterial community composition among the four groups and used PCoA ordination to visualize the differences graphically. Multivariate logistic regression analysis after adjusting for possible confounding factors is performed using the glm function in R to ascertain the significant influences for chronic diseases and is calculated as adjusted odds ratio (95%, confidence interval [CI]). Linear discriminant analysis (LDA) coupled with the effect size (LEfSe) algorithm was used to identify biomarkers that were characteristics of each group based on the abundance values (Segata et al., 2011).

Continuous data were expressed as median, and dichotomous variables as counts and proportions. Chi-square test was used to compare categorical data. Group wise comparisons of abundance of various taxa in the different groups were performed using Kruskal-Wallis H test and Wilcoxon rank sum test (for pairwise comparisons). The Benjamini–Hochberg FDR method was used to correct the multiple comparisons (Benjamini and Hochberg, 1995). P-values < 0.05 were considered statistically significant. The data visualization in this study was mostly conducted in R environment using ggplot2 (Wickham, 2017). All statistical analyses were performed using R 4.0.

### Availability of Data and Materials

The 16S rRNA sequencing data were deposited in the NCBI’s Sequence Read Archive (SRA) database under the accession ID PRJNA673060. unique persistent identifier and hyperlink to dataset(s) in https://www.ncbi.nlm.nih.gov/bioproject/673060.

## Results

### Characteristics and Clinical Information of the Participants

A total of 2507 adult healthy subjects were included in the present study. The MR group consisted of 538 adults (231 male) with a median age of 53 years; the MU group consisted of 529 adults (179 male) with a median age of 49 years; the PR group consisted of 765 adults (274 male) with a median age of 53 years, and the PU group included 675 adults (232 male) with a median age of 55 years (**Table 1**).

**Figure 2:**
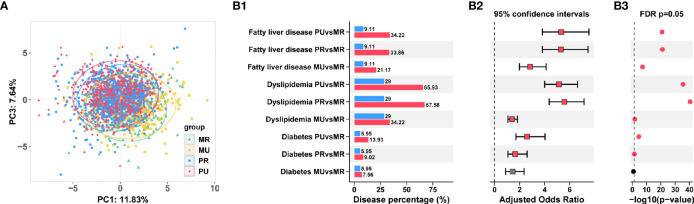
Prevalence of chronic diseases in the epidemiological investigation among the four groups in Ningxia Province China. **(A)** Separation of clinical variables among the MR, MU, PR, and PU groups revealed by Principal component analysis (PCA). **(B)** Prevalence of diabetes, dyslipidemia, and fatty liver disease in the four groups **(B1)**. The adjusted Odds ratios **(B2)** and FDR adjusted p-values were also shown **(B3)**.

Participant details and clinical baseline information were shown in **Table S1**. PCA analysis demonstrated that the four groups were well separated (**Figure 2A**), suggesting a recognizable alteration in clinical variables.

Consistently, we found that with the deepening of urbanization (MR→MU→PR→PU), the prevalence of chronic diseases had a gradually increasing trend, such as diabetes, dyslipidemia, and fatty liver disease. The highest prevalence among chronic diseases was dyslipidemia, followed by fatty liver disease. Further logistics regression analysis revealed that the risk of chronic diseases was significantly higher in the MU, PR, and PU groups compared with the MR group (odds ratio significantly greater than 1) after adjusting for gender and age effects (**Figure 2B** and **Table S2**).

### Dietary Information and Gut Microbial Diversity Analysis

**Figure 3:**
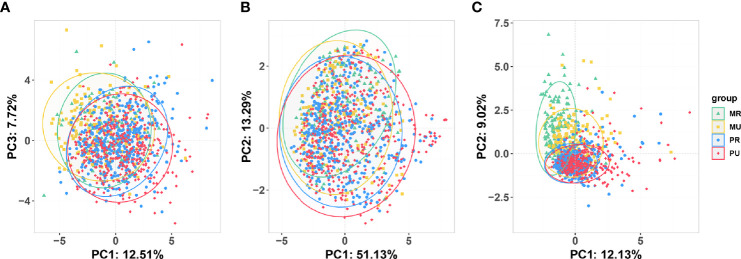
Overview of the unconstrained (Unsupervised) ordination analysis of the participants who had undergone 16S rRNA testing for gut microbiota. **(A)** Principal component analysis (PCA) of clinical variables (1087 samples). **(B)** Principal coordinate analysis based on Bray-Curtis distance shows beta-diversity (similarity between 1177 samples) among the four groups. **(C)** PCA of dietary metadata (1045 samples). MR, mountain rural; MU, mountain urban; PR, plain rural; PU, plain urban.

With the development of urbanization, people’s lifestyle and dietary habits have changed. To detect changes in diet and gut microbiota, we screened the 2,507 participants and obtained stool samples from 1,177 people (1,045 of whom had dietary questionnaire information). Separation of clinical metadata among the four groups (1087 samples) was revealed by PCA (**Figure 3A** and **Table S3**). Similarly, significant shift was also observed on the four groups in the overall composition of the gut microbiota at genus level as indicated by PCoA (**Figure 3B** **)** and PERMANOVA analysis (**Table S4**). Coherently, the PCA results revealed that the four groups were significantly different in dietary habits (**Figure 3C**). The MU, the PR and the PU groups consumed more meat, rice, and fewer potatoes than the MR group (**Table S5**).

### Taxonomic Composition and Diversity of Fecal Microbiota

**Figure 4:**
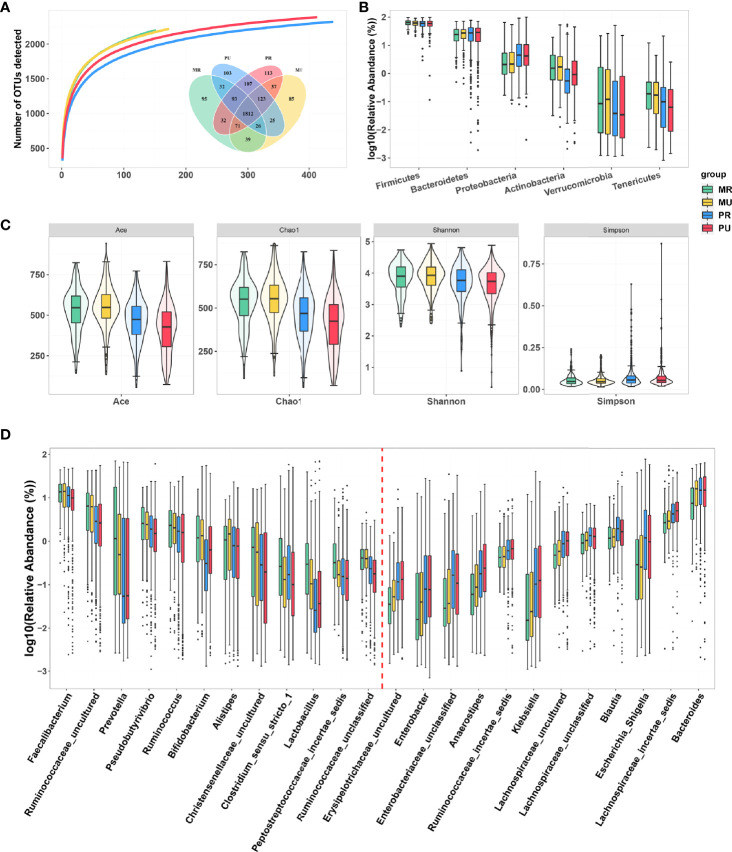
Gut microbial diversity of participants in the MR, MU, PR, and PU groups. **(A)** The rarefaction analysis between the number of samples and the number of OTUs. As the number of samples increased, the number of OTUs approached saturation in MR (n = 152), MU (n = 173), PR (413) and PU (439) groups. A Venn diagram displaying the overlaps between groups showed that 1892 of the total number of 2793 OTUs were shared in the four groups. **(B)** Taxonomic composition of the gut bacteria on the phylum level of the MR, MU, PR, and PU groups. The gut microbiota of the four groups are dominated by *Firmicutes*, followed by *Bacteroidetes*, *Actinobacteria* and *Proteobacteria*. **(C)** Diversity estimation of the 16S ribosomal RNA gene library of the MR, MU, PR, and PU groups. MR, Mountain rural; MU, Mountain urban; PR, Plain rural; PU, Plain urban; OTUs, operational taxonomic units; PCoA, principal coordinate analysis. **(D)** The significantly different bacterial genera based on Wilcoxon rank-sum test in the four groups. With the deepening of urbanization, the abundance of some bacterial genera decreased (left box) and some increased (right box) as shown by red line delimited.

In the four groups, the rarefaction analysis showed that the number of OTUs richness nearly approached saturation as the number of samples increased (**Figure 4A**). A Venn diagram displaying the overlaps among groups showed that 1812 of the total number of 2793 OTUs were shared in the four groups (**Figure 4A**), indicating a high similarity in the core microbiota across the four regions. Overall, the fecal microbiomes across the four groups were dominated by *Firmicutes* followed by *Bacteroidetes*, *Actinobacteria* and *Proteobacteria* (**Figure 4B**). Bacterial members from Verrucomicrobia and Tenericutes were also detected in most of the subjects, although their abundance was observed to be low (< 0.02%) (**Table S6**). *Firmicutes* and *Bacteroidetes* abundance did not change significantly among the four groups, but the abundance of *Proteobacteria* was higher, while the abundance of *Actinobacteria* was lower in the MU, the PR and the PU groups compared with the MR group (**Table S6**). Furthermore, the microbial community richness indicated by Ace and Chao 1 estimators and community diversity estimated by Shannon and Simpson indices showed significant changes among the four groups (**Figure 4C** and **Table S7** **)**. Namely, the microbial community gut richness and diversity were significantly lower in the PR and PU groups compared with the MR and the MU groups (**Figure 4C** and **Table S7**).

### Variations in Gut Microbial Community Across Regions

Overall, at the genus level, the top ten genera among the four groups were *Bacteroides, Faecalibacterium*, *Ruminococcaceae_uncultured*, *Prevotella, Lachnospiraceae_incertae_sedis, Subdoligranulum, Escherichia-Shigella, Pseudobutyrivibrio, Ruminococcus*, and *Blautia* (**Table S8**). The differences in taxonomic composition are also manifested at the genus level. Differential analysis revealed that with the deepening of urbanization (MR→MU→PR→PU), some bacterial genera, such as *Faecalibacterium, Prevotella, Pseudobutyrivibrio, Ruminococcus*, and *Bifidobacterium* gradually decreased, while some bacterial genera, such as *Bacteroides, Blautia, Klebsiella, Anaerostipes*, and *Enterobacter* gradually increased (**Figure 4D** and **Table S8**). These results are consistent with PCoA (**Figure 3B**) and PERMANOVA analysis (**Table S4**), indicating that region have significant influence on the microbial community profiles of the gut microbiota.

### Effect Size and Correlation Analysis

**Figure 5:**
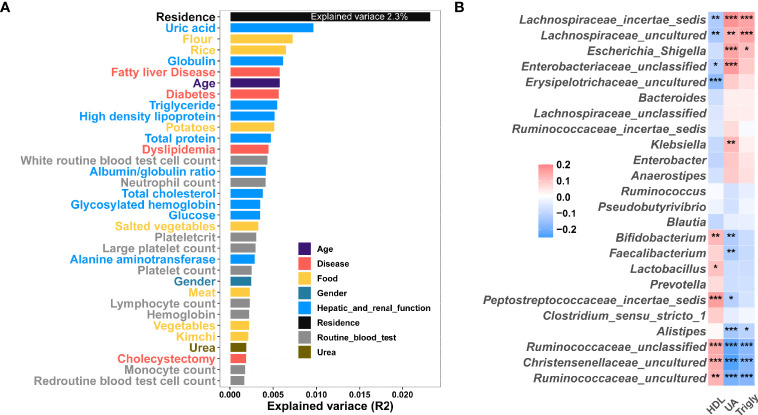
Correlation between clinical indexes, diet and gut microbiota. **(A)** Effect size of clinical indexes and diet, which contribute significantly to the variance (R^2^) of the fecal microbiota (Adonis p < 0.05). This analysis was based on all subjects (1177 samples). **(B)** Heatmap showing the Correlation between clinical variable and microbiome. The colors of the cell indicate the Spearman correlation coefficients among the 24 OTUs in Fig. 4 and 3 clinical variable high-density lipoprotein (HDL), uric acid (UA), and triglyceride (Trigly). The significance level in the correlation test is denoted as: *q < 0.05, **q < 0.01; ***q < 0.001.

In order to investigate the effect of external factors on gut microbiota, we performed effect size and correlation analysis. In this study, we showed that residence was an important explanatory variable for fecal microbiota composition along the urban-rural gradient, as it explained 2.3% of the variance (**Figure 5A**). In total, clinical metadata and diet explained 18.7% of the variance in the gut microbiota (**Figure 5A**). Furthermore, diet explained 2.9% of the variance, while hepatic and renal function explained 5% of the variance and blood routine explained 2.5% of the variance (**Figure 5A** and **Table S9**). To further explore the links between gut microbiota, and clinical variables, we performed a Spearman correlation analysis. It was found that the bacteria such as *Ruminococcaceae_uncultured*, *Ruminococcaceae_unclassified*, *Christensenellaceae_uncultured*, *Faecalibacterium* and *Bifidobacterium*, enriched in the MR group were positively correlated with HDL and negatively correlated with UA and Triglyceride, while bacteria enriched in the PU group, such as *Lachnospiraceae_incertae_sedis*, *Lachnospiraceae_uncultured*, *Escherichia-Shigella*, and *Enterobacteriaceae_unclassified*, were negatively correlated with HDL and positively correlated with UA and Triglyceride (**Figure 5B**).

### Region-Specific Signatures in Gut Microbial Composition

**Figure 6:**
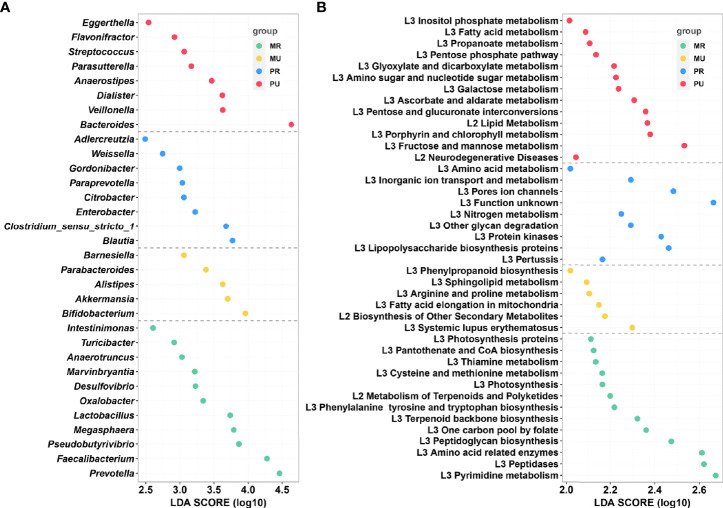
LEfSe analysis and Kyoto Encyclopedia of Genes and Genomes (KEGG) assignments of the mountain rural (MR), mountain urban (MU), plain rural (PR), and plain urban (PU) groups. **(A)** LEfSe analysis identified the predominant taxons of each group. Green represents the bacteria specific for the MR group, yellow represents the bacteria specific for the MU group, blue represents the bacteria specific for the PR group, and red represents the bacteria specific for the PU group. **(B)** Presentation of the altered KEGG pathways in each group. Grouping was performed in the same way as in Figure **(A)** LDA scores were calculated by LDA effect size, using the linear discriminant analysis to assess effect size of each differentially abundant bacterial taxa **(A)** or KEGG pathways **(B)**.

To further identify the region-specific species signatures, investigation was performed at the genus level using LEfSe analysis. A total of 32 genera were identified as the sets of region-specific. Based on LDA selection, the MR group was significantly enriched with 11 genera including *Faecalibacterium*, *Prevotella*, and *Pseudobutyrivibrio*, the MU group was significantly enriched with 5 genera including *Bifidobacterium*, *Akkermansia*, and *Alistipes*; the PR group was significantly enriched with 8 genera including *Blautia*, *Clostridium_sensu_stricto*_1, and *Enterobacter*, and the PU group was significantly enriched with 8 genera including *Bacteroides*, *Veillonella*, *Dialister*, and *Anaerostipes* (**Figure 6A**).

### Region Specific Trends in Functional Profile

Generally, for healthy individuals, differences at the taxonomic level the gut microbiomes may show remarkably similar profile in functional repertoire. Therefore, to investigate the region-specific functional characteristics of the current microbiome dataset, we attempted to investigate region-specific trends in the functional composition of the gut microbiomes of the four groups. The KEGG orthology (KO) and the KEGG pathway/module profile were constructed using the PICRUSt version 1.0.0 pipeline and the 16S rRNA marker gene sequences were used to predict the microbial community function profiles. Different groups were observed to have distinct trends in their functional profiles of the gut microbiomes. The NSTI values per sample calculated by PICRUSt were in the range of 0.018-0.29 (**Table S10**). As shown in **Figure 6B**, KEGG pathway analysis found 41 significantly enriched pathways related to metabolism and disease in the four groups. Based on the LDA selection, 13 predicted microbial functions including terpenoid backbone biosynthesis, metabolism of terpenoids and polyketides, and thiamine metabolism were remarkably enriched in the MR group, 6 functions including fatty acid elongation in mitochondria and sphingolipid metabolism were remarkably enriched in the MU group, 9 predicted microbial functions including lipopolysaccharide biosynthesis proteins and glycan degradation were remarkably enriched in the PR group, and 13 predicted microbial functions including fatty acid metabolism, lipid metabolism, fructose and mannose metabolism, and propanoate metabolism were remarkably enriched in the PU group (all p < 0.05, **Figure 6B** and **Table S11**).

## Discussion

In the 21st century, with the rapid development of the world economy, there is an emerging consensus that urbanization is affecting human health despite its economic importance and contribution to development. Diet, air and water contamination, socioeconomic status, and other environmental factors have long-term effects on human intestinal microbiota that may increase the risk of chronic diseases during urbanization. The adoption of Western lifestyle, diet and exposure to higher levels of pollution might have led to the loss of specific bacterial species of our ancestral microbiota, which in turn increased the prevalence of chronic diseases (Ng et al., 2015). Urbanization may alter the gut microbiome and further affect residents’ health, but studies are currently lacking. For the first time, we studied the increase in the prevalence of chronic diseases during rapid industrialization in Ningxia, China, and significant differences in gut microbiota and function between rural and urban areas within populations of homogeneous ethnicity. The disparate patterns of the gut microbiota composition in rural and urban areas offer an opportunity to understand the contribution of a “rural microbiome” in potentially protecting against the development of chronic diseases.

Our results revealed that *Firmicutes* and *Actinobacteria* gradually decreased while *Proteobacteria* gradually increased as urbanization deepened (MR→MU→PR→PU). This is consistent with the literature where the predominant microbial populations in rural populations were from the *Firmicutes* and *Actinobacteria* phyla (Tyakht et al., 2013). These bacterial communities are favored by the consumption of starch-rich bread and potatoes, typical foods for rural Russia and some low-income socioeconomic groups (Jahns et al., 2003; Tyakht et al., 2013; Tyakht et al., 2014). It was reported that urbanization is associated with an increased proportion of *Bacteroides*, *Blautia*, *Faecalibacterium*, *Ruminococcus*, and *Bifidobacterium*, whereas *Prevotella* is increased in the gut microbiota of individuals residing in non-industrialized societies (De Filippo et al., 2010; Yatsunenko et al., 2012; Schnorr et al., 2014). These findings suggest that the urbanization process has significantly altered the gut microbiota composition of genetically similar populations living in different regions, such as rural versus urban. Alterations of the *Prevotella : Bacteroides* ratio have been associated with certain diet. High intake of amino acids, lipids, and cholesterol has been shown to increase the abundance of *Bacteroides*, while *Prevotella* favors the intake of sugars and other complex carbohydrates (De Filippo et al., 2010; Wu et al., 2011; Schnorr et al., 2014). Our findings showed that with the progress of urbanization, *Faecalibacterium*, *Prevotella* gradually decreased while *Bacteroides* increased. Children from rural African villages with high fiber intake have abundant *Prevotella*, which has the ability to hydrolyze cellulose and xylan (De Filippo et al., 2010). In general, gut microbiota adapt to a polysaccharide-rich diet, enabling the host to extract energy from dietary fiber while protecting against inflammation (Sonnenburg and Sonnenburg, 2014). This may be related to the low prevalence of chronic diseases, as we found a significant enrichment of *Faecalibacterium* in the MR group. *Faecalibacterium* is one of the most abundant bacterial genera in the adult colon of healthy individuals, accounting for more than 5% of the total bacterial population (Miquel et al., 2013; Miquel et al., 2014). *Faecalibacterium* is known for its immunomodulatory properties and is effective in improving intestinal inflammation and intestinal barrier function (Sokol et al., 2008; Martín et al., 2015; Miquel et al., 2015; Quévrain et al., 2015).

Our results demonstrated that urban residents consumed more meat and less whole grains, which were similar to Western dietary habits. Western diet is mainly characterized by consumption of more high saturated fat, red meat and carbohydrates, and less fresh fruits, vegetables and whole grains. In mouse and human studies, Western diet have been shown to influence the pathogenesis and/or development of many diseases, including diabetes, obesity, hypercholesterolemia, and cardiovascular disease (Ley et al., 2008; Turnbaugh et al., 2008; Conlon and Bird, 2014; Agus et al., 2016; Carrillo-Larco et al., 2016). In addition, we also found that with the deepening of urbanization, the diversity of gut microbiota also gradually decreased, which may also be related to diet. Individuals who consume foods based on raw plants or fiber have a higher diversity of gut microbiota than westerners on Western diet (De Filippo et al., 2010; Yatsunenko et al., 2012; Schnorr et al., 2014; Martínez et al., 2015; Obregon-Tito et al., 2015). Similarly, animal experiments have shown that a low-fiber diet is associated with depletion of microorganisms in mice, becoming more aggressive and irreversible over several generations (Agus et al., 2016). These data suggested that Western diet were associated with irreversible gut microbiota dysbiosis and partially explained the impact of industrialization on microbiome alterations.

Furthermore, urban residents are more exposed to food additives, which can also cause changes in gut microbiota. For example, artificial sweeteners can induce dysglycaemia by altering the gut microbiota, reinforcing the glycan degradation capacity of gut microorganisms and increasing energy harvesting and a number of pathways involved in sphingolipid metabolism and transport (Suez et al., 2014). Consistently, our study found that sphingolipid metabolism was enriched in the MU group and glycan degradation was enhanced in the PR group. It should be pointed out that in this study, clinical and dietary metadata explained only 18% of the gut microbiota variance, indicating that there are other factors that affect gut microbiota, such as air quality. Air pollution coincides with urbanization and is believed to have an increasing adverse impact on public health (Gong et al., 2012), including an increase in cardiovascular and respiratory diseases (Franklin et al., 2015; Samet and Gruskin, 2015), and gastrointestinal disease (Kaplan et al., 2010; Ananthakrishnan et al., 2011). Alternatively, as shown in **Figure 5**, bacterial communities were also influenced by host factors. Thus, MaAsLin was used to analyze associations of microbial taxa with age and gender. Preliminary results revealed that *Lachnospia*, *Akkermansia*,and *Escheichia-Shigella* were associated with age, while *Phascolarctobacteium* was associated with gender (data not shown). The relevance of these bacteria to disease will be further explored in the future.

In summary, consistent with the theory of microbiota disappearance and its association with the emergence of chronic diseases, a loss of gut bacterial richness and diversity during urbanization was observed, which may be associated with the high prevalence of chronic diseases. The limitations of this study are based on 16S rRNA gene sequencing, which may provide incomplete and biased insight into the composition of intestinal microbiota. An in-depth understanding of intestinal bacterial species or strains during urbanization and their function in the pathogenesis of chronic diseases is still needed.

## Conclusion

Our findings suggest that the gut microbial composition and function, which are influenced by residence and diet, differ according to the level of urbanization. Therefore, the increase in the prevalence of chronic diseases that accompanies urbanization is associated with aberrant in the composition of gut microbiome. The reduction in microbial diversity based on 16S rRNA sequences simply indicated an unbalanced gut ecosystem, but did not provide us with more detailed information about strains (species) or certain microbial functions. Overall, functional studies are also lacking. Since reducing the incidence of chronic diseases and guaranteeing the health of residents are crucial in terms of economic and social development, we propose that it is necessary to establish a focused and novel framework for the study of specific roles of gut microbiomes in the pathogenesis of chronic diseases during urbanization at a fine scale.

## Data Availability Statement

The datasets presented in this study can be found in online repositories. The names of the repository/repositories and accession number(s) can be found below: https://www.ncbi.nlm.nih.gov/, PRJNA673060.

## Ethics Statement

This study was approved by the Institutional Review Board of the General Hospital of Ningxia Medical University (No. 2017-200). All the participants provided written informed consent before completing the survey and health examination. The patients/participants provided their written informed consent to participate in this study.

## Author Contributions

Conception and design of the study: YD and LD. Collection of samples: LN, TS, BW, SZ, CY, and XL. Epidemiological investigation: XS, LW, SH, and ZW. Laboratory test of serum and urine: FL, XG, and YZ. Ultrasound examination: JT. Analysis and interpretation of data: LD, TS, and XS. Drafting of the manuscript: YD and LD. Administrative support and study supervision: YD and ZHL. All authors read, revised, and approved the final draft.

## Funding

The study was supported by the Research and Development Plan of the 13th Five-Year Plan of Ningxia Hui Autonomous Region (the major S&T projects. Grant No. 2016BZ02) and The First-Class Discipline Construction Project in Colleges and Universities of Ningxia (Grant No. NXYLXK 2017A05).

## Conflict of Interest

The authors declare that the research was conducted in the absence of any commercial or financial relationships that could be construed as a potential conflict of interest.

## Publisher’s Note

All claims expressed in this article are solely those of the authors and do not necessarily represent those of their affiliated organizations, or those of the publisher, the editors and the reviewers. Any product that may be evaluated in this article, or claim that may be made by its manufacturer, is not guaranteed or endorsed by the publisher.

## Abbreviations

MR, Mountain rural; MU, Mountain urban; PR, Plain rural; PU, Plain urban; LDL, Low density lipoprotein; HDL, High density lipoprotein; TC, Total cholesterol; TG, Triglyceride; UA, Uric acid; CT, Computed tomography; OTUs, Operational Taxonomy Units; IBD, Inflammatory Bowel Disease; PcoA, Principal coordinate analysis; PCA, Principal Component Analysis; CI, Confidence interval; LDA, Linear discriminant analysis; LefSe, Linear discriminant analysis coupled with the effect size; FDR, False Discovery Rate; SRA, Sequence Read Archive.
